# Disability pension by occupational class - the impact of work-related factors: The Hordaland Health Study Cohort

**DOI:** 10.1186/1471-2458-11-406

**Published:** 2011-05-30

**Authors:** Inger Haukenes, Arnstein Mykletun, Ann Kristin Knudsen, Hans-Tore Hansen, John Gunnar Mæland

**Affiliations:** 1 Department of Public Health and Primary Health Care, University of Bergen, Kalfarveien 31, NO-5018 Bergen, Norway; 2 The Norwegian Institute of Public Health, Nydalen, N-0403 Oslo, Norway; 3 Department of Health Promotion and Development, University of Bergen, Christiesgt. 13 NO-5020 Bergen, Norway; 4 Department of Sociology, University of Bergen, Rosenbergsgt. 39, NO-5020 Bergen, Norway

## Abstract

**Background:**

The social gradient in disability pension is well recognized, however mechanisms accounting for the gradient are largely unknown. The aim of this study was to examine the association between occupational class and subsequent disability pension among middle-aged men and women, and to what extent work-related factors accounted for the association.

**Methods:**

A subsample (N = 7031) of the population-based Hordaland Health Study (HUSK) conducted in 1997-99, provided self-reported information on health and work-related factors, and were grouped in four strata by Erikson, Goldthorpe and Portocareros occupational class scheme. The authors obtained follow-up data on disability pension by linking the health survey to national registries of benefit (FD-trygd). They employed Cox regression analysis and adjusted for gender, health (medical conditions, mental health, self-perceived health, somatic symptoms) and work-related factors (working hours, years in current occupation, physical demands, job demands, job control).

**Results:**

A strong gradient in disability pension by occupational class was found. In the fully adjusted model the risk (hazard ratio) ranged from 1.41 (95% CI 0.84 to 2.33) in the routine non-manual class, 1.87 (95% CI 1.07 to 3.27) in the skilled manual class and 2.12 (95% CI 1.14 to 3.95) in the unskilled manual class, employing the administrator and professional class as reference. In the gender and health-adjusted model work-related factors mediated the impact of occupational class on subsequent disability pension with 5% in the routine non-manual class, 26% in the skilled manual class and 24% in the unskilled manual class. The impact of job control and physical demands was modest, and mainly seen among skilled and unskilled manual workers.

**Conclusions:**

Workers in the skilled and unskilled manual classes had a substantial unexplained risk of disability pension. Work-related factors only had a moderate impact on the disability risk. Literature indicates an accumulation of hazards in the manual classes. This should be taken into account when interpreting the gradient in disability pension.

## Background

Being employed contrary to unemployed is highly valued in modern society and it is associated with good health [1]. However, among those employed, the risk of exclusion from working life as a result of disability pension varies considerably by education, occupation and income. Compared with the extensive body of literature concerning the social gradient in health, studies addressing the gradient in disability pension are limited but the findings are consistent. Non-medical factors such as social background from childhood, low level of education, low occupational status and low income seem to be strong determinants of disability pension in both genders [2-5].

Differences in work-related exposure levels between occupational classes are well recognised [6-9]. Niedhammer et al. [7] found a strong gradient by occupational class when examining exposure levels for adverse physical, ergonomic and chemical working environments. As regards the psychosocial working environment, a low level of job control was frequently reported in both non-manual and manual classes, while high job demands were more common in higher-ranking white collar occupations. However, high exposure levels in the work environment do not necessarily predict future disability pension. A review by Allebeck & Mastekaasa [10] found limited scientific evidence for the impact of physically demanding work on disability pension and moderate evidence for low job control. More recent studies report that both low job control and high physical demands are associated with subsequent disability pension [11-13]. In addition, part-time work, high work-unit aggregated job strain, unfavourable ergonomic and physical working environment and shift work have been added to the list of risk factors [14-17].

A limited number of longitudinal studies have employed the Erikson, Goldthorpe and Portocareros occupational class scheme (EGP scheme) when examining social differences in disability pension [4,5]. The EGP scheme is constructed by combining information on occupation, employment, education (skilled versus unskilled), industry (manual versus non-manual) and size of company [6], and has been recommended by the World Health Organization (WHO) to overcome differences in classification between studies of different populations [18]. Employing the EGP scheme, Krokstad et al. [4] found a strong gradient in disability pension by occupational class, and that low job control and manual work increased the risk in all classes. However, research within this field is scarce. There is a need for prospective, population based studies that examine the mediating role of work-related factors in the relation between occupational class and disability pension.

The present study has two aims: first, to examine the association between occupational class (EGP scheme) and subsequent disability pension in a middle-aged Norwegian population and second, to examine the extent to which work-related factors account for this association.

## Methods

### Population and data material

The Hordaland Health Study (HUSK) was conducted during the period 1997-1999 in Hordaland County in western Norway. HUSK was a collaboration between the National Health Screening Service, the University of Bergen and local health services. The study population included all individuals living in Hordaland County born during the period 1953-1957 (29,400), aged 40-45 years at the time of the health study. A total of 8,598 men and 9,983 women participated, yielding a participation rate of 57% for men and 70% for women, and 63% in total. Data collection was performed in two steps. Firstly, all participants underwent a physical health examination and completed a self-administered questionnaire. Secondly, the participants were randomised in four equal groups (two male and two female groups). Each of these groups was given a gender-specific questionnaire.

The sub-sample used in the current study (3,548 men and 5,348 women) answered a questionnaire containing the Swedish Demand-Control-Support Questionnaire (DCSQ) [19].

We included participants with valid scores on the demand and control subscales in DCSQ who reported more than 100 paid working hours during the preceding year (n = 7244). This procedure led to an exclusion of 1,652 participants. Further, self-employed farmers and agricultural labourers were excluded due to limited numbers (n = 201). Finally, individuals awarded disability pensions in the period from participation in HUSK and 12 months ahead were excluded (n = 12). In total we excluded 1865 (21%) participants, 613 (17%) men and 1,252 (23%) women. The total study sample consists of 7,031 individuals (2,935 men and 4,096 women), i.e. 79% of the sub-sample who answered the DCSQ.

### Outcome

The outcome was the awarding of disability pension during follow-up, from one year after participating in the heath survey (HUSK) until the end of 2004. Using personal ID numbers, which are issued to all Norwegians at birth, the health survey was linked to national benefit registers (FD-trygd) by Statistics Norway. The registers contain records of monthly payments of disability pension, and the accuracy is well documented [20]. For all disability pensioners, the time interval between the date of participation in HUSK and the date of the disability pension award was calculated. A wash-out period of 12 months after the date of participation was established in order to eliminate report bias as a result of participants already being in the process of applying for a disability pension.

### Occupational class

The Erikson, Goldthorpe and Portocareros social class scheme (EGP scheme) [21] was used to classify participants into occupational classes. The EGP scheme has achieved a high degree of comparability when applied in order to measure morbidity differences by occupational class in different European countries [22]. In the current study, self-reported information on branch of industry and occupation/occupational title was manually converted into four-digit codes based on the International Standard Classification of Occupations, ISCO-88 (COM) [23]. Using an internationally applicable algorithm by Ganzeboom and Treiman [24], ISCO codes were firstly recoded into a seven-class EGP scheme [6,22]. The seven classes were: higher administrators and professionals (I), lower administrators and professionals (II), routine non-manual workers (III), skilled manual workers (V+VI), unskilled manual workers (VIIa), self-employed farmers (IVc), and agricultural labourers (VIIb). Secondly, skilled and unskilled manual workers were redistributed according to their educational level. Workers reporting lower secondary school or lower (≤10 years) as their highest educational levels were classified as unskilled, and the rest were classified as skilled [24]. In addition, we chose to merge higher and lower administrators and professionals (I+II) and excluded self-employed farmers (IVc) and agricultural labourers (VIIb) due to limited numbers. The four occupational classes used are shown in Table 1.

**Table 1:** Distribution of participants, incidence of disability pension awards, total and stratified by gender.

				Disability pension		
				
Occupational class	Total n (%)	Men n (%)	Women n (%)	Total n (%)	Men n (%)	Women n (%)
				p < 0.001*	p = 0.003*	p < 0.001*
Administrators and professionals	2393 (34.0)	1442 (49.1)	951 (23.2)	32 (1.3)	17 (1.2)	15 (1.6)
Routine non-manual workers	2492 (35.4)	444 (15.1)	2048 (50.0)	73 (2.9)	9 (2.0)	64 (3.1)
Skilled manual workers	1535 (21.8)	780 (26.6)	755 (18.4)	62 (4.0)	23 (2.9)	39 (5.2)
Unskilled manual workers	611 (8.7)	269 (9.2)	342 (8.3)	42 (6.9)	11 (4.1)	31 (9.1)

Total	7031 (100)	2935 (100)	4096 (100)	209 (3.0)	60 (2.0)	149 (3.6)

*χ^2 ^for differences between groups

### Work-related factors

Experience of job demands and job control were measured using the Swedish Demand-Control-Support Questionnaire (DCSQ) [19], a 17-item questionnaire developed by Theorell [25] based on the Demand-Control model [26]. The demand subscale has five items, four of which measure level of work pace (whether the job requires you to work very fast, very hard, with too great effort, and whether you have sufficient time), while one measures occurrences of conflicting demands. The control subscale has four items measuring the level of skill discretion (whether your have an opportunity to learn new things, be creative and utilise skills and competence) and two items measuring decision authority (the authority to decide what work should be carried out and how). Because of a translation error when translating the Swedish version of the questionnaire into Norwegian, the control item addressing competence had to be excluded in the analyses. However, the psychometric properties of the 16-item Norwegian version of the DCSQ are found to be satisfactory, also in groups with low education [19].

In multivariate analyses, mean scores for the demand and control subscales were categorised into five groups (the 20^th^, 40^th^, 60^th ^and 80^th ^percentiles) to pick up the underlying variation. The lowest percentile (20^th^) represents low demand and high control, respectively.

Physical demands at work were self-reported in four categories (sedentary, walking, walking and lifting, heavy manual work) [27]. The number of paid working hours per week were categorised into four groups (> 40, 37-40, 20-36, < 20 hours), and years in current occupation into five groups (< 5, 5-10, 11-15, 16-20, > 20 years).

The distribution of work-related factors by occupational class was categorised as mean scores in tertiles on the subscales of job demand and job control, where the highest tertile was used to characterise high demands by occupational class and low control by occupational class. Tertiles were employed due to an empirical testing of the relation between the cumulative incidence of disability pension and levels of job demands and job control. For control we found a substantial increase in disability pension equivalent with the highest tertile. For demands the increase was limited.

Physical demands were dichotomised into low level (sedentary and walking) and high level (walking/lifting and heavy manual work), the number of paid working hours per week into part-time (< 37 hours) and full-time (≥37 hours), and years in current occupation into more or less than the mean years (≤14 years and > 14 years).

### Health

Somatic diagnoses were assessed by self-reported occurrence of coronary infarction, stroke, diabetes, asthma, multiple sclerosis, chronic bronchitis, osteoporosis and fibromyalgia. Information about the number of somatic diagnoses was computed as a continuous variable. Further information about somatic conditions was obtained by assessing whether the participants had used any medication the previous day, and, if so, for which condition. Based on this information, an appropriate ICPC diagnosis was made by a team of physicians. A continuous variable indicating the number of somatic conditions for which the person was taking medication was then established [28].

Mental health was assessed by the Hospital Anxiety and Depression Scale (HADS) [29].

Scores were used as continuous variables reflecting the symptom load of anxiety and depression [29].

Self-perceived physical and mental health status was measured by the self-report Short Form-12 (SF-12) [30]. This shorter version of the SF-36 is recommended for large population surveys such as HUSK [30]. Weighted summation provides summary scores for perceived mental health and perceived physical health. Out of the total of 12 questions in SF-12, eight assess the level of limitations due to perceived mental or physical health. The measurement has been standardised in accordance with the US norm data [30] with a mean score of 50 (SD 10). Somatic symptom load was estimated by asking participants about the presence of 17 commonly experienced symptoms from different organ systems [31]. Answers on a five-point Likert scale were summed up and used as a continuous variable, with increasing levels reflecting a higher symptom load [29].

### Analysis

Differences in the distribution of disability pension by occupational class, disability pension by work-related factors and work-related factors by occupational class were tested using Chi-square tests. Potential gender differences were examined by performing stratified Chi-square tests.

Cox proportional hazards models were employed to estimate the relationship between occupational class and subsequent disability pension. The administrators and professionals were used as reference group, and the results are presented as gender-adjusted hazard ratios (HR) with 95% confidence intervals (CIs).

The regression analyses were conducted stepwise, and both separate and cumulative adjustments were performed. In the cumulative model, the variables were added in a predefined order. Firstly, health variables were introduced to avoid overestimation of the mediating effect of work-related factors introduced later in the model. Health variables were introduced en bloc (somatic diagnosis, somatic conditions based on medication use, mental health, self-perceived health and somatic symptoms). Secondly, we adjusted for years in current occupation and thirdly, working hours per week. In a forth step, we added physical demands, thereafter job demands and, finally, job control.

The impact (%) of each work-related factor (or set of factors) on the occupational gradient in disability pension represents the change in HR when extending the model with a new variable. The following formula was used: (HR _extended _- HR _initial_)/HR _initial _*100. In separate adjustments, the initial model is the gender-adjusted one. In cumulative adjustments, the initial model is the step preceding each extended step.

The analyses were performed using SPSS 15.0 for Windows.

### Ethical approval

The study protocol was approved by the Regional Committee for Medical Research Ethics, Western Norway and by the Norwegian Data Inspectorate. Written statements of informed consent were gathered from all the participants in the current study at the time of the physical health examination.

## Results

Among the participants, men were most likely to work as administrators and professionals, and women in occupations classified as routine non-manual. Only small gender differences were seen among skilled and unskilled manual workers (Table 1).

A total of 209 (2.9%) of the participants were awarded a disability pension during follow-up. For both genders, the cumulative incidence of disability pension increased from the administrators and professionals to the unskilled manual workers (Table 1). The differences in disability pension between the occupational classes were significant for both men and women.

Low job control and high physical demands were most often reported among skilled and unskilled manual workers and least often among administrators and professionals, while high job demands showed a slightly inverse distribution (Table 2). For all work-related factors, the differences in distribution by occupational class were significant (Table 2).

**Table 2:** Distribution of work-related factors by occupational class, stratified by gender.

	Work-related factors									
	
	**Part-time work**^**a**^		**Years in current occupation**^**b**^		**High physical demands**^**c**^		**High job demands**^**d**^		**Low job control**^**e**^	
	
	Men	Women	Men	Women	Men	Women	Men	Women	Men	Women
Occupational class	(%)	(%)	(%)	(%)	(%)	(%)	(%)	(%)	(%)	(%)
	p < 0.001*	p < 0.001*	p < 0.001*	p = 0.001*	p < 0.001*	p < 0.001*	p < 0.001*	p < 0.001*	p < 0.001*	p < 0.001*
Administrators and professionals	(6.3)	(35.0)	(58.9)	(47.5)	(7.9)	(10.6)	(47.9)	(45.1)	(19.6)	(22.0)
Routine non-manual workers	(13.5)	(50.8)	(52.6)	(46.3)	(12.0)	(18.4)	(39.9)	(34.9)	(37.6)	(49.4)
Skilled manual workers	(17.7)	(73.6)	(71.8)	(48.3)	(51.5)	(59.4)	(29.9)	(32.1)	(46.3)	(62.3)
Unskilled manual workers	(15.8)	(69.7)	(71.4)	(35.8)	(58.3)	(46.0)	(35.3)	(30.1)	(62.8)	(80.7)

* χ^2 ^for differences between groups

^a ^Less than 37 hours per week

^b ^More than 14 years (mean) in current occupation

^c ^Walking/lifting and heavy manual work

^d ^Highest tertile of mean scores in tertiles, on demand sub-scale

^e ^Highest tertile of mean score in tertiles, on control sub-scale

### Work-related factors and disability pension

Decreasing levels of job control and increasing levels of physical demands were related to a higher incidence of disability pension, while increasing levels of job demands were not (Table 3). Fewer working hours per week was also associated with a higher incidence of disability pension. The differences in the incidence of disability pension between strata of job control, physical demands and working hours per week were significant for both genders. The association between increasing years in current occupation and higher rates of future disability pension was significant for men only.

**Table 3:** Distribution of participants and incidence of disability pension in strata of work-related factors.

		Disability pension		
		
		Total	Men	Women
Work-related factors	n (%)	n (%)	n (%)	n (%)
Working hours per week		p < 0.001*	p < 0.001*	p = 0.034*
> 40	576 (8.2)	11 (1.9)	8 (1.8)	3 (2.3)
37-40	3825 (56.3)	88 (2.3)	33 (1.6)	55 (3.2)
20-36	1949 (28.7)	73 (3.7)	11 (3.8)	62 (3.7)
< 20	440 (6.5)	30 (6.8)	5 (16.1)	25 (6.1)
				
Years in current occupation		p = 0.241*	p = 0.017*	p = 0.266*
< 5	1198 (17.6)	26 (2.2)	6 (1.6)	20 (2.4)
5-10	1130 (16.6)	37 (3.3)	6 (1.6)	31 (4.1)
11-15	1266 (18.6)	40 (3.2)	7 (1.4)	33 (4.4)
16-20	1693 (24.9)	46 (2.7)	10 (1.3)	36 (3.8)
> 20	1507 (22.2)	54 (3.6)	29 (3.4)	25 (3.7)
				
Physical demand		p < 0.001*	p < 0.001*	p < 0.001*
Mainly sitting	2987 (45.3)	53 (1.8)	21 (1.4)	32 (2.1)
Sitting and standing	1946 (29.5)	57 (2.9)	10 (1.6)	47 (3.5)
Walking and lifting	1504 (22.8)	73 (4.9)	18 (3.5)	55 (5.6)
Hard manual work	151 (2.3)	8 (5.3)	8 (5.8)	0 0
				
Job demand		p = 0.213*	p = 0.754*	p = 0.211*
1 (low level)	1030 (14.6)	23 (2.2)	6 (1.8)	17 (2.5)
2	1377 (19.6)	33 (2.4)	8 (1.4)	25 (3.1)
3	1057 (15.0)	35 (3.3)	10 (2.3)	25 (4.0)
4	1798 (25.6)	56 (3.1)	18 (2.3)	38 (3.8)
5 (high level)	1769 (25.2)	62 (3.5)	18 (2.3)	44 (4.5)
				
Job control		p < 0.001*	p = 0.024*	p < 0.001*
1 (high level)	1173 (16.7)	21 (1.8)	10 (1.6)	11 (2.0)
2	948 (13.5)	11 (1.2)	6 (1.3)	5 (1.0)
3	1964 (27.9)	37 (1.9)	12 (1.4)	25 (2.3)
4	859 (12.2)	40 (4.7)	11 (3.4)	29 (5.4)
5 (low level)	2087 (29.7)	100 (4.8)	21 (3.2)	79 (5.5)

*χ^2 ^for differences between groups.

### Risk of disability pension by occupational class

The risk (HR) of disability pension by occupational class followed an occupational gradient in all steps of the regression analyses (cumulative adjustments, Table 4). In the fully adjusted model, skilled and unskilled manual workers had twice as high risk of subsequent disability pension than administrators and professionals (table 4). The reduction in hazard ratios from the gender-adjusted model to the fully adjusted model was 20% for non-manual workers, 35% for skilled manual workers and 55% for unskilled manual workers. Adjusting for health in the first step led to a 42% reduction in hazard ratio among unskilled manual workers, and a smaller reduction in the remaining classes.

We found no interaction effects between gender and occupational class (p > 0.05).

**Table 4:** Risk of disability pension by occupational class, separate and cumulative adjustments.

					Work-related factors									
					
Separate adjustments	Gender only		**Health**^**a**^		Years in current occupation		Working hours per week		Physical demands		Job demands		Job control	
**Occupational class**	**HR**	**95% CI**	**HR**	**95% CI**	**HR**	**95% CI**	**HR**	**95% CI**	**HR**	**95% CI**	**HR**	**95% CI**	**HR**	**95% CI**

Administrators and professionals	1.00		1.00		1.00		1.00		1.00		1.00		1.00	
Routine non-manual workers	1.76	(1.15-2.71)	1.49	(0.95-2.36)	1.73	(1.12-2.67)	1.74	(1.11-2.72)	1.65	(1.05-2.59)	1.86	(1.21-2.87)	1.51	(0.96-2.35)
Skilled manual workers	2.86	(1.86-4.38)	2.53	(1.60-3.98)	2.58	(1.66-3.99)	2.88	(1.84-4.50)	2.24	(1.38-3.63)	3.08	(2.00-4.73)	2.26	(1.44-3.55)
Unskilled manual workers	4.76	(3.00-7.55)	2.78	(1.65-4.68)	4.71	(2.95-7.51)	4.74	(2.93-7.67)	3.93	(2.35-6.57)	5.20	(3.26-8.28)	3.39	(2.07-5.56)
														
**Cumulative adjustments**	**Gender only**		**+ health**^**a**^		**Years in current ** **occupation**^**b**^		**+ Working hours ** **per week**^**c**^		**+ Physical demands**^**d**^		**+ Job demands**^**e**^		**+ Job control**^**f**^	

Administrators and professionals	1.00		1.00		1.00		1.00		1.00		1.00		1.00	
Routine non-manual workers	1.76	(1.15-2.71)	1.49	(0.95-2.36)	1.55	(0.98-2.46)	1.58	(0.98-2.54)	1.51	(0.93-2.45)	1.52	(0.93-2.47)	1.41	(0.84-2.33)
Skilled manual workers	2.86	(1.86-4.38)	2.53	(1.60-3.98)	2.44	(1.53-3.87)	2.57	(1.58-4.17)	2.21	(1.30-3.76)	2.20	(1.29-3.74)	1.87	(1.07-3.27)
Unskilled manual workers	4.76	(3.00-7.55)	2.78	(1.65-4.68)	2.78	(1.64-4.71)	2.95	(1.71-5.09)	2.57	(1.43-4.62)	2.60	(1.44-4.67)	2.12	(1.14-3.95)

Hazard ratios (HRs) and 95% CIs from Cox regression models adjusted for gender.

^a ^Adjusted for gender and health (somatic diagnosis, somatic conditions based on medication use, mental health, self-perceived health and somatic symptoms)

^b ^Adjusted for gender, health and years in current occupation

^c ^Adjusted for gender, health, years in current occupation and working hours per week

^d ^Adjusted for gender, health, years in current occupation, working hours per week and physical demands

^e ^Adjusted for gender, health, years in current occupation, working hours per week, physical demands and job demands

^f ^Adjusted for gender, health, years in current occupation, working hours per week, physical demands, job demands and job control

### The impact of work-related factors on the gradient in disability pension

The combined impact of work-related factors, i.e. from the gender and health-adjusted model to the final model, was 5% for non-manual workers, 26% for skilled manual workers and 24% for unskilled manual workers (cumulative adjustments, Table 4).

In the separate adjustment model, job control mediated the impact of occupational class on disability pension by 14% among non-manual workers, 21% among skilled manual workers and 29% among unskilled manual workers (Table 4). In the cumulative model, (final step) the impact was reduced to 7% among non-manual workers, 15% among skilled manual and 18% among unskilled manual workers (Table 4). In the separate adjustment model, physical demands accounted for 6% of the reduction in disability risk among routine non-manual workers, 22% among skilled manual workers and 17% among unskilled manual workers. When physical demands were introduced in the cumulative model (step 5), the impact decreased to 4% among non-manual workers and 13-14% among manual workers. Years in current occupation mainly influenced the disability risk among skilled manual workers (10%), but less impact was seen when entered in the cumulative model (step 3). Working hours per week had no impact in the separate model, but caused a slight increase in the hazard ratios in the cumulative model (step 4). Job demands increased the hazard ratio in the non-manual and manual classes, but in the cumulative model the impact was insignificant.

## Discussion

### Main results

We found substantial differences in disability pension by occupational class, with a twofold risk of subsequent disability pension among skilled and unskilled manual workers compared with administrators and professionals. The excess risk was found after adjusting for health and work-related factors. Work-related factors had a moderate impact on the risk of disability pension among skilled and unskilled manual workers and a limited impact among workers in routine non-manual occupations.

### Strengths

The present study was based on a linkage between the Hordaland Health Study Cohort and the National Insurance Administrations records of disability pension awarded to Norwegian inhabitants from 1992 onwards. The register is complete, accurate and independent of exposure data obtained in the Hordaland Health survey. The study thus avoids the problem of attrition. The study design excluded individuals awarded disability pension up to 12 months after baseline, thus decreasing the risk of biased information from participants in the process of being awarded disability pension [1]. The follow-up period was one to seven years, a period without major changes in Norwegian disability policy with the potential to alter the cumulative incidence by occupational class. Moreover, new incidences of awards in this age group have been relatively stable during this period. With respect to health and work-related factors, most of them were measured using questionnaires with well-documented psychometric properties. Self-reported exposure data at baseline were collected without participants or administrators being aware of future research hypotheses.

### Limitations

There are some limitations to our study. First, healthy worker selection in manual occupations may have caused an underestimation of the disability pension risk. Second, non-participation was probably more frequent among individuals with a low educational level and low income [32]. This may have caused a variation in the response rate across occupational classes and may have biased the strata comparability. A previous study of the HUSK population also found poorer health status among non-participants in the health survey, which may further increase the healthy worker selection effect [33].

Third, the health study provided little information about exposure linked to specific occupations. Information of this kind may have contributed to explaining more of the work-related disability risk among manual workers [13,16].

Furthermore, events during follow-up that may have influenced the outcome, such as a job change or sudden health problems, were not controlled for. As regards job changes, workers reporting the most unfavourable working conditions (unskilled manual workers) could be expected to be more inclined to change jobs than their colleagues. Although a change of job is possible, it is less likely that this has led to a change in occupational class. Changing jobs within an occupational class could alter the experience of work-related factors, but probably not substantially.

Moreover, the EGP scheme has been criticised for not being adapted to major changes in class structure, including the rapid growth in female work participation[34]. In our study, half of the female participants and only a small proportion of the men were classified as routine non-manual workers. Although heterogeneous as regards occupation and educational level, the majority in this class is employed in the public sector and performs interactive service work. However, the heterogeneity could conceal sub-class differences in health and disability pension.

Further, the list of somatic diagnosis is restricted, and a more extensive list may have increased the impact of health in the regression analyses.

Finally, baseline data on health and work-related factors are cross-sectional, which prevents us from testing causality between work-related factors and health on the path to disability pension. Introducing work-related factors in a health-adjusted model probably underestimates the impact of work-related factors on disability pension, since such factors may cause health problems.

### Interpretation of the disability gradient

#### Health selection

Childhood medical history can contribute to occupational stratification and also influence recruitment to disability pension [35]. In the present study, health had a substantial impact on the risk of disability pension among unskilled manual workers, indicating a relationship between prior health condition and the position as unskilled in the labour market. This may further confound the association between occupational class and disability pension. On the other hand, hazards in the working environment most prevalent in manual occupations could mediate the impact of occupational class on subsequent disability pension [11,13,16].

The occupational gradient could also be explained by health-selective mobility. However, studies indicate that mobility between classes serves more to dilute health differences than to heighten them [36]. This dilution may arise because the risk of illness in 'the class of origin' seems to follow a person to 'the class of destination' [37].

#### Incentive hypothesis

The incentive hypothesis states that individuals voluntarily decide to leave the labour market because the alternative of social benefits is perceived as equal to or more gainful than work [38]. In Norway, benefit levels are high, while differences in income across occupational classes are moderate. For unskilled workers with low income, a relatively high compensation rate could influence thresholds for considering disability pension as a solution to health problems, and thereby influence the gradient.

#### The impact of job control

The association between job control and subsequent disability pension has been found in population-based studies and studies of industry, as well as of specific occupations [9,11,16,39,40]. In a population-based study, decision authority accounted for 10-13% of disability pension cases in both genders after adjusting for age, smoking, BMI and ergonomic working environment exposure [11]. Furthermore workers in the construction industry who reported low job control had a significantly increased risk (odds ratio = 1.86) of receiving disability pension [16]. Among nurses, low influence at work was associated with subsequent disability pension in a model adjusting for income [40]. However, contradictory findings have also been reported [41-43]. In a population-based cohort study, no support was found for the impact of decision authority on subsequent disability pension [41].

In the present study, we found that job control had a moderate impact on the occupational gradient in disability pension. Considering the high proportion of manual workers who reported low control, the relatively modest impact on subsequent disability pension was surprising. One explanation may be that the theoretical basis for Erikson, Goldthorpe and Portocareros' occupational class scheme is the neo-Weberian tradition. In this tradition, one criterion for distinguishing between occupations was authority and control in the work process [6]. Considering this, it is possible that the impact of low job control is underestimated due to the occupational class scheme used in this study.

An additional explanation is that workers in mid-life with long service in the same branch of industry have become familiar with a work situation characterised by low control. In this case, the reporting of low job control would simply be a description of how work is performed and not signal a poor working environment that could influence disability pension. On the other hand, consistent reporting of low job control indicates a working environment in which learning to utilise one's own skills and take responsibility for decisions is given little priority. A poor learning environment at work may have a negative transfer value to other arenas of life, where learning to achieve control is important in relation to maintaining good health.

Moreover, low job control could influence workers' opportunities to protect themselves against work-related health hazards, thereby increasing the disability risk [11].

#### The impact of physical demands

The limited contribution of physical demands to the occupational gradient in disability pension was even more surprising. With few exceptions, the literature is consistent as regards the predictive value of physically demanding work in relation to disability pension [9,13,16,43]. In a population-based study, Labriola et al. [13] found that physically demanding work was significantly associated with disability pension (hazard ratio = 1.83) after adjusting for health behaviour and psychosocial work factors. Studies in industries and occupations characterised by manual work have also found a strong association between unfavourable ergonomic working conditions and disability pension [16]. A similar association was found in nursing, a highly female-dominated occupation [40]. However, Albertson et al. [41] did not find any impact of physically demanding work on subsequent disability pension.

The limited impact of physical demands on subsequent disability pension in the present study may be explained by a strong healthy worker effect. A Danish study [9] found that high physical demands predicted subsequent unemployment and early retirement, but were not associated with disability pension. Among middle-aged construction workers, low job control and high physical demands were associated with low self-reported work ability at baseline, but had limited impact on disability pension during follow-up [42]. These studies indicate that workers who experience high physical demands may seek other solutions than disability pension if they experience health problems or impaired ability to work.

## Conclusion

In conclusion, our study found a substantial unexplained risk of disability pension among workers in skilled and unskilled manual occupations compared with workers in administrative and professional occupations. Although the majority of the workers in skilled and unskilled manual occupations reported low job control and high physical demands, the impact on the gradient in disability pension was modest. However, major differences in work-related exposure between occupational classes, point to a possible accumulation of hazards among manual workers that should be taken into account when interpreting the gradient in disability pension.

## Competing interests

The authors declare that they have no competing interests.

## Authors' contributions

IH conceived of the study, performed the data analysis, drafted the manuscript and coordinated the study. AM participated in conceiving the study, advised the statistical analyses, interpretation of the results and drafting the manuscript. AKK advised the statistical analyses and revised the manuscript for important content. HTH revised the manuscript for important content. JGM participated in conceiving the study, interpretation of the results and drafting the manuscript. All authors read and approved the final manuscript.

## Funding

None.

## Pre-publication history

The pre-publication history for this paper can be accessed here:

http://www.biomedcentral.com/1471-2458/11/406/prepub
